# Vasoreparative Dysfunction of CD34^+^ Cells in Diabetic Individuals Involves Hypoxic Desensitization and Impaired Autocrine/Paracrine Mechanisms

**DOI:** 10.1371/journal.pone.0093965

**Published:** 2014-04-08

**Authors:** Yagna P. R. Jarajapu, Sugata Hazra, Mark Segal, Sergio LiCalzi, Chandra Jhadao, Kevin Qian, Sayak K. Mitter, Mohan K. Raizada, Michael E. Boulton, Maria B. Grant

**Affiliations:** 1 Department of Pharmaceutical Sciences, College of Pharmacy, Nursing, and Allied Sciences, North Dakota State University, Fargo, North Dakota, United States of America; 2 Departments of Pharmacology and Therapeutics, College of Medicine, University of Florida, Gainesville, Florida, United States of America; 3 Department of Nephrology, College of Medicine, University of Florida, Gainesville, Florida, United States of America; 4 Department of Ophthalmology, Indiana University School of Medicine, Indianapolis, Indiana, United States of America; 5 Department of Physiology and Functional Genomics College of Medicine, University of Florida, Gainesville, Florida, United States of America; Bristol Heart Institute, University of Bristol, United Kingdom

## Abstract

We hypothesized that endothelial progenitor cells derived from individuals with diabetes would exhibit functional defects including inability to respond to hypoxia and altered paracrine/autocrine function that would impair the angiogenic potential of these cells. Circulating mononuclear cells isolated from diabetic (n = 69) and nondiabetic (n = 46) individuals were used to grow endothelial colony forming cells (ECFC), early endothelial progenitor cells (eEPCs) and isolate CD34^+^ cells. ECFCs and eEPCs were established from only 15% of the diabetic individuals tested thus directing our main effort toward examination of CD34^+^ cells. CD34^+^ cells were plated in basal medium to obtain cell-free conditioned medium (CM). In CM derived from CD34^+^ cells of diabetic individuals (diabetic-CM), the levels of stem cell factor, hepatocyte growth factor, and thrombopoietin were lower, and IL-1β and tumor necrosis factor (TNFα) levels were higher than CM derived from nondiabetic individuals (nondiabetic-CM). Hypoxia did not upregulate HIF1α in CD34^+^ cells of diabetic origin. Migration and proliferation of nondiabetic CD34^+^ cells toward diabetic-CM were lower compared to nondiabetic-CM. Attenuation of pressure-induced constriction, potentiation of bradykinin relaxation, and generation of cGMP and cAMP in arterioles were observed with nondiabetic-CM, but not with diabetic-CM. Diabetic-CM failed to induce endothelial tube formation from vascular tissue. These results suggest that diabetic subjects with microvascular complications exhibit severely limited capacity to generate ex-vivo expanded endothelial progenitor populations and that the vasoreparative dysfunction observed in diabetic CD34^+^ cells is due to impaired autocrine/paracrine function and reduced sensitivity to hypoxia.

## Introduction

Many diabetic individuals with ischemic cardiac and vascular disease remain symptomatic despite exhausting conventional medical therapy and mechanical revascularization. Increasing evidence suggests that microvascular insufficiency plays a significant role in the pathophysiology of this ischemia. Recognizing the magnitude of this problem, investigators have worked to develop new treatments that have led to the evolution of therapeutic angiogenesis.

Preclinical and clinical data provide evidence that growth factors and stem/progenitor cells may be used therapeutically for repair of ischemic tissue. Preclinical studies have provided evidence for safety and the potential therapeutic potency of vascular progenitor cells. Clinical trials using a variety of approaches have supporting the feasibility, safety and bioactivity of these cells for treatment of advanced cardiovascular disease with the goal of repairing ischemic tissue.

While the majority of clinical studies that are currently being undertaken involve the use of CD34^+^ cells, culture-derived cells such as endothelial colony-forming cells (ECFC) and early endothelial progenitors (eEPC) may represent an alternative for vascular repair [1]–[3]. The cell surface marker CD34^+^ distinguishes a progenitor population with marked clinical utility [4], [5]. In individuals with diabetes, circulating CD34^+^ cell numbers predict cardiovascular dysfunction and risk better than CD34^+^VEGFR2^+^- and CD133^+^-based populations [6], [7]. Fadini et al [8] reported that circulating CD34^+^ cell numbers represented an independent risk biomarker of cardiovascular events and significantly correlated with outcomes in metabolic syndrome.

ECFC are “true” endothelial progenitor as the cells become endothelial cells and form capillaries as tested by the in vivo matrigel assay in SCID mice [9]. In addition to their angiogenic properties in vitro and in vivo, these cells can be differentiated by cell surface markers. ECFCs express the endothelial markers CD31, CD141, CD105, CD146, CD144, vWF, flk-1, and to a lesser extent, the progenitor cell markers CD133, CD34, and CD117. On the other hand, eEPCs also known as endothelial cell-colony forming cells (CFU-ECs), have myeloid progenitor cell activity, differentiate into phagocytic macrophages, and are not vasculogenic [3] but have shown in vivo efficacy for vascular repair by promoting revascularization via paracrine mechanisms [10]–[12].

Recent studies have shown that autologous cells derived from diabetic patients are not as effective at tissue repair as those from nondiabetic or healthy volunteers [13]–[17]. As many cardiovascular disease patients who are candidates for cell therapy have diabetes, understanding the optimal stem/progenitor population to use is imperative. Thus, while individuals with diabetic complications represent a population that may greatly benefit from cellular therapy, their broadly dysfunctional cells limit the feasibility of an autologous cellular approach [18]. Diabetes is associated with a reduced number of circulating progenitor cells, [19] and the cells demonstrate reduced proliferative potential, impaired migratory and generalized vasoreparative functions in *in vivo* models of vascular injury [14], [15], [20], [21].

The degree of vascular engraftment by the cell populations used for vascular repair ranges from none to a modest percentage [15], [20], [22], [23]. These differences may be due to the degree of injury and/or the particular vascular bed examined. However, in most cases, the increase in neovascularization/revascularization is not related to the precise number of cells that differentiate into endothelial cells, but rather to the paracrine effects provided by these cells. The cytokines and growth factors released from these cells stimulate resident endothelium to accelerate vascular repair [20], [23], [24]. Despite this, little is known about the secretome of endothelial progenitors and how this impacts the reparative capabilities in particular in diabetes.

Ramos et al [25] demonstrated that cord blood CD34^+^CD45^+^CD133^+^CD38^+^ cells injected into the mouse hind limb ischemia (HLI) model did not integrate into the host vasculature; however, they successfully restored revascularization and blood flow. Forty-eight hours after local injection of CD34^+^ cells into ischemic murine limbs, blood flow increased compared to untreated limbs [20]; however, maximal effects were observed weeks later, when injected cells were no longer detected, [20] emphasizing the significance of their lingering paracrine modulatory effects.

A previous study by Majka et al [26] reported that human CD34^+^ cells release numerous growth factors and cytokines. Harraz et al [23] showed that CD34^−^CD14^+^ or CD34^−^ cells can incorporate into the endothelium of blood vessels in mouse ischemic limbs only in the presence of CD34^+^ cells, indicating paracrine modulation of CD14^+^ cells by CD34^+^ populations. Krenning et al [27] reported that CD34^+^ cells modulate proliferation and endothelial differentiation of CD14^+^ cells by releasing HGF, interleukin (IL)-8, and monocyte chemoattractant protein-1 (MCP-1). Recently, CD133^+^ cells were shown to secrete interleukins, growth factors, and chemokines that are capable of accelerating vascular network formation *in vivo* and enhancing healing of ischemic ulcers in diabetic mice[28]. Culture-derived progenitor cells release IL-8, producing a mitogenic effect on vascular endothelial cells [29]. Paracrine factors released by these cells confer cerebrovascular protection by increasing prostacyclin (PGI_2_) production via COX2/PGI_2_ synthase and by reducing thromboxane A_2_. [30] Furthermore, these cells express cyclooxygenase (COX)-1 and secrete PGI_2_, increasing angiogenesis via activation of PPARδ [31].

The reparative ability of progenitors is largely due to their capacity to home to areas of hypoxia. Thus, the sensitivity of CD34^+^ cells to hypoxia plays a critical role in initiating the vasoreparative function of these cells. Hypoxic/ischemic environments release stromal cell-derived factor (SDF) and VEGF, and progenitor cells can only respond to these signals if they express CXCR4, VEGFR1, and VEGFR2 [32], [33]. Furthermore, hypoxic preconditioning enhances the vasoreparative function of progenitor cells in both *in vitro* and *in vivo* angiogenesis assays[34], an effect that may be due to increased CXCR4, VEGFR1, and VEGFR2 surface expression.

In this study, we tested the hypothesis that diabetic individuals with microvascular complications would exhibit reduced ability to establish ex vivo expanded populations (eEPC and ECFC) and that diabetes alters the sensitivity to hypoxia, secretory profile and autocrine/paracrine functions of CD34 cells.

## Methods

### Ethics statement

The Institutional Review Board (IRB) and ethics committee of Gainesville Health Science Center at University of Florida and Shands Hospital at University of Florida and of Indiana University, Indianapolis have specifically approved this study [Approved protocols: IRB 2010-163 and 2011-407(University of Florida); IRB # 1308124973 (Indiana University)]. Informed consent was obtained from study subjects after explanation of the nature and possible consequences of the study in accordance with the approved IRB protocols. All the participants provided their written consent to participate in the study. The consent procedure was approved by the IRB (IRB 2010-163, IRB 2011-407 and IRB # 1308124973) and participants' written consents were documented. This entire study was conducted in the United States of America.

### Patient Characteristics

We examined ECFCs as potential sources of vascular progenitors for autologous cell therapy. Peripheral blood was obtained from a cohort of subjects with Type 2 diabetic (T2D) (cohort 1, Table 1; n = 20) for isolation of ECFCs. Blood samples were always obtained between 1PM and 5 PM. Individuals were nonsmokers, were treated with oral hypoglycemic agents and were not taking insulin. Patient were not on statins, as they had no overt cardiovascular disease (CVD) all LDL-C <100 mg/dL (2.6 mmol/L). Eight of 17 patients had documented BP ≥140/80 mm Hg prior to initiation of antihypertensive therapy and were followed in Endocrinology Clinic at University of Florida. The treatment strategy was in accordance with ADA guidelines and focused on life style changes as well as pharmacotherapy with ACEI (n = 6) or ARB (n = 2). These patients on treatment had BP of <130/80 mm Hg. These same 8 patients also had modest (30–299 mg/day [microalbuminuria]) or high (≥300 mg/day [macroalbuminuria or clinical albuminuria]) urinary albumin excretion and one of the 8 individuals had nephropathy. Individuals were 30–69 years of age. There was a slight but not significant increase in the HgbA_1C_ of the 17 diabetic individuals from which ECFC could not be grown. All 20 selected diabetic subjects were free of concurrent illness or other diseases known to influence CD34^+^ cell function. Exclusion criteria were acute infection, immune diseases, current or past hematologic disorders or malignancy, drug-induced diabetes mellitus, unstable angina, recent (within 6 months) myocardial infarction or stroke, liver failure, dialysis because of renal failure, pregnancy, history of organ transplant, presence of graft and lack of consent to participate in the study. Table 1 illustrates main clinical data of the 20 subjects who were the participants in this first study.

**Table 1:** Characteristics of Diabetic Individuals Used for ECFCs and CFU-ECs.

	Diabetic (ECFCs)	Diabetic (No ECFCs)
Number	3	17
Gender M/F	2/1	5/12
Age	60±12	53±16
HbA1C	7.6±1.1	8.1±1.7
Retinopathy	2	3
Neuropathy	1	4
Nephropathy	-	1
Hypertension	-	8
Hypercholesterolemia	-	0

For the second cohort of subjects, the characteristics of nondiabetic and diabetic individuals are shown in Table 2. ND (n = 45) or DM (n = 54) individuals aged 32–60 years old were recruited from University of Florida clinics and blood samples were always obtained between 1PM and 5 PM. Individuals were nonsmokers. Type 1 diabetic (T1D) individuals (n = 13) were treated insulin and T2D individuals (n = 41) were treated with oral hypoglycemic agents and were not taking insulin. Forty three diabetic individuals were not on statins, as they had no overt CVD all LDL-C <100 mg/dL (2.6 mmol/L). Six individuals were on high dose statins therapy with goals achieved of LDL-C <100 mg/dL (2.6 mmol/L). Seven patients had documented BP ≥140/80 mm Hg prior to initiation of antihypertensive therapy and pharmacotherapy with ACEI (n = 5) or ARB (n = 2) and achieved treatment blood pressure of <130/80 mm Hg. Five individuals (N = 2 T1D and N = 3 T2D) of these 7 individuals had diabetic nephropathy. As in cohort all 54 selected diabetic subjects in cohort 2 were free of concurrent illness and met the inclusion criteria. Three T2D individuals had distal symmetric polyneuropathy. All diabetic individuals had nonproliferative diabetic retinopathy but no individual had proliferative diabetic retinopathy.

**Table 2:** Characteristics of Control and Diabetic Individuals.

	Control	Diabetic
Number	45	54
Gender M/F	17/14	21/13
Age	50±4	55±4
HbA1C	4.9±0.2	7.5±0.3 (P<0.0001)

		Type 1 (13)	Type 2 (41)
Retinopathy		12	37
Neuropathy		0	3
Nephropathy		2	3
Hypertension		2	5
Hypercholesterolemia		0	6

Exclusion criteria: Evidence of ongoing acute or chronic infection (HIV, hepatitis B or C, tuberculosis), ongoing malignancy, cerebral vascular accident or cerebral vascular procedure, history of organ transplantation, presence of a graft, uremic symptoms, an estimated glomerular filtration rate of <40 cc/min, or an albumin of less than 3.6.

### Cell Isolation and Conditioned Medium (CM)

Peripheral blood mononuclear cells (MNCs) were enriched for CD34^+^ cells by immunomagnetic selection (Easysep, Human CD34 positive selection kit, StemCell Technologies, Inc.) as per supplier's instructions.[14] Briefly, total monocytes from peripheral blood were obtained by gradient centrifugation using ficoll reagent (Ficoll-Paque, GE Healthcare Biosciences). Plasma was completely excluded from the cell fraction by a series of washings using phosphate-buffered saline with 2% fetal bovine serum (FBS) and 1 mM EDTA. These cells were enriched for lineage negative cells by using a negative selection kit (StemCell Technologies, Inc.) as per supplier's instructions. Lineage negative cells were further enriched for CD34^+^ cells by using a positive selection kit. CM was prepared by suspension culture of cells in endothelial basal medium (Lonza), for 18 hours in round bottom 96-well plates at a density of 20,000 cells/150 μl/well. Medium was collected by centrifugation at 440×g for 10 minutes to remove cells and debris and concentrated 10× using CentriPrep filtering units (Amicon Ultra-15 Millipore centrifugal filter units, 3 kDa cut-off) at 4°C. CM was snap-frozen in liquid nitrogen and preserved at −80°C until analysis was performed. Basal medium was concentrated and preserved in a similar manner as the control medium where applicable. CM concentrates were used for quantitative analysis, and for experiments determining reactivity of intact arteries. However, unconcentrated CM was used for evaluating proliferation, cAMP and cGMP generation in intact arteries, and for evaluating their effect on angiogenesis. Quantitative analysis of selected cytokines and growth factors (Table 3) in the CM was performed by AssayGate Inc. using the Luminex Bead-based immunoassay platform.

**Table 3:** Concentration of cytokines and growth factors in the conditioned medium (CM) from nondiabetic and diabetic CD34^+^ cells.

Factor Analyzed	Nondiabetic	Diabetic	N	P<
Stem cell factor	18560±1132	4594±758	6	0.0001
Hepatocyte growth factor (HGF)	117±25	13±2	6	0.006
Monocyte chemoattractant protein-1 (MCP1)	1420±426	2058±761	6	NS
Granulocyte-colony stimulating factor (GCF)	2±0.8	30±10	6	NS
Monocyte chemoattractant protein-1 (MCP1)	1420±426	2058±761	6	0.03
Granulocyte monocyte-colony stimulating	5±1	18±4	6	0.03
Factor (GM-CSF)				
FMS-like tyrosine kinase-3 ligand (Flt3L)	4313±836	6029±1215	5	NS
Thrombopoietin (TPO)	56±6	5±1	5	0.0002
Erythropoietin (EPO)	ND	ND	6	
Angiopoietin-1 (Ang)	1082±270	734±61	5	NS
Interleukin (IL)-1β	14±3	46±13	6	NS
IL-3	240±59	371±97	5	NS
IL-6	553±160	2870±312	6	0.001
IL-8	2709±651	5857±1785	6	NS
IL-10	18±4	55±17	6	NS
Tumor necrosis factor (TNF)-α	129±35	34±9	5	0.23
TNF-β	ND	ND		
Transforming growth factor (TGF)-β1	671±220	185±65	6	NS
TGF-β2	15±4	5±1	6	NS
TGF-β3	ND	ND	6	
Decorin	62±15	4±0.9	6	0.02
Basic nerve growth factor (bNGF)	ND	ND		
Insulin-like growth factor (IGF)-1	110±13	659±197	6	0.03
IGF binding protein (IGFBP)-3	35480±623	89280±10570	6	0.008

Concentrations (pg/ml) were expressed as Mean ± S.E.M. ND – not detected; NS – not significant.

### Culturing ECFCs and eEPCs

For ECFCs, peripheral blood MNCs were suspended in EBM-2 medium supplemented with EGM-2MV single quotes (Lonza). 10 million cells from both healthy and diabetic donor were plated on each well of a 6 well plate. The plates were coated with rat tail collagen, type-1 (BD Biosciences, Bedford, MA). The cells were allowed to attach for 4 days, and after 4 days, the medium was changed every other day for 3–4 weeks.

For eEPCs, peripheral blood MNCs were suspended in EBM-2 medium supplemented with EGM-2MV single quotes. Ten million cells from healthy and diabetic donors were plated on each well of a 6 well plate. The plates were coated with fibronectin (Sigma, St. Louis, MO) at least an hour prior to plating the cells. After 96 hours, the non-adherent cells were collected and were plated in new fibronectin-coated 6 well plates. The medium was changed at day 4 and day 7. The adherent spindle shaped cells have appeared between 7–10 days. Images were obtained when cells were almost 100% confluent by light microscopy with 50× magnification.

### Exposure of Cells to Hypoxia

The pO_2_ environment was regulated by an oxygen-controlled glove box attached via a central airlock with O_2_, CO_2_, N_2_, temperature, and humidity controls (Coy Laboratory Products Inc., Grass Lake, MI).[35] Hypoxia was created by maintaining pO_2_ of 5 mmHg. Culture medium StemSpan was pre-equilibrated for 24 hours prior to beginning any experiment. Cells were incubated at 37°C for 4 hours and then subjected to either flow cytometry or western blotting, as described below. Changes in HIF1α due to exposure to hypoxia were further confirmed by using dimethyloxalylglycine (N-(methoxyoxoacetyl)-glycine methyl ester (DMOG) that mimics intracellular hypoxia by increasing HIF1 α expression by inhibiting prolyl hydroxylase. [36] Cells were treated with 150 μM CoCl_2_ for 12 hours or 1 mM DMOG for 24 hours in StemSpan.

### Western Blot Analysis of HIF-1α

Equal concentrations of proteins from whole cell lysates were resolved by SDS-PAGE, blotted onto a nitrocellulose membrane and stained with rabbit polyclonal anti human antibodies to HIF-1α (Abcam). Blots were processed by using ECL Plus (GE Healthcare). GAPDH was used as a loading control. Band intensities were quantified using Image J software (NIH).

### Flow Cytometric Analysis of VEGFR1, VEGFR2, and CXCR4 Receptor Expression

To assess the surface expression of receptors, CD34^+^ cells from diabetics and non-diabetics were treated with FcR blocking reagent (anti-CD16/32, TruStain, BioLegend) and then stained with Zenon Alexa Fluor 488-(Invitrogen) tagged primary mouse monoclonal anti-human VEGFR1, antihuman VEGFR2, and CXCR4 antibodies (R&D systems), or isotype control antibodies for 20 minutes on ice. Cells were analyzed by a FACS Scan flow cytometer (Becton Dickinson).

### Cell Proliferation and Migration Assays

Proliferation was determined by division tracking cytometry using a CellTrace CFSE (carboxyfluorescein diacetate, succinimidyl ester) proliferation kit (Invitrogen) as per the manufacturer's instructions. CFSE-loaded CD34^+^ cells derived from nondiabetic individuals were incubated with CM for 72 hours. Fluorescence was quantified by flow cytometry and analyzed by grouping into three different regions based on the fluorescence intensity. Non-proliferating cells retain higher amounts of CFSE while proliferation results in lower amounts. Up to 10,000 events were recorded per sample and percent gated events in different regions were compared among different groups. Proliferation was further confirmed by evaluating BrdU incorporation (Cell Proliferation ELISA; Roche Bioscience) as per the manufacturer's instructions. The assay was performed using 10,000 cells treated with CM for 48 hours.

Migration was determined by the QCM™ chemotaxis 5 μm 96-well cell migration assay (Chemicon International, Inc.) as per the manufacturer's instructions in CD34+ cells derived from nondiabetic individuals. Migratory response to SDF-1α or CM was expressed as percent increase in migrated cells compared to untreated cells.

### Studies in Isolated Arteries

Animal procedures were reviewed and approved by the Institutional Animal Care and Use Committee (IACUC) of the University of Florida (200902518). The investigation conformed to the *Guide for the Care and Use of Laboratory Animals* (NIH Publication No. 85–23, revised 1996).

Rat (male Sprague-Dawley, 250–300 g) cerebral arteries were studied as described previously.[37] Posterior cerebral arteries (PCAs) were isolated and cannulated with glass pipettes mounted in an arteriograph (Living Systems Inc., Burlington, VA) with oxygenated physiological saline solution (PSS) for diameter measurements. PSS or PSS containing 1×10^5^ cells was perfused into the lumen under no-flow conditions so that the arterial segment was exposed intraluminally to the cells or the vehicle at 37°C. After 1 hour, intraluminal pressure was increased slowly from 10 to 100 mm Hg in increments of 30 mm Hg. Constriction to pressure (myogenic tone) was calculated according to the following equation:
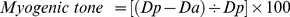
(1)where Da is the internal diameter of the arterial segment with active myogenic tone in the presence of PSS, and Dp is the passive diameter obtained with the exposure to calcium-free PSS.

Effects of nondiabetic-CM or diabetic-CM were evaluated in mesenteric arterial segments in a small vessel wire-myograph (Danish MyoTechnology) and normalized as previously described.[38] Segments were pre-incubated with CM for 30 minutes before evaluating reactivity to phenylephrine or relaxation to bradykinin following pre-constriction to phenylephrine (0.5 ml of CM was added to the bath containing 5 ml of PSS). Contraction or relaxations before and after treatment with CM were compared.

Assays of cGMP and cAMP generated by CM in intact mesenteric arterial segments were performed using a DiscoverX HitHunter assay as per manufacturer's instructions (GE Healthcare Biosciences).

### *In Vitro* Angiogenesis

The angiogenic effect of CM was evaluated *in vitro* in the microvascular tissue derived from mouse corpus cavernosum cultured in matrigel (growth factor-reduced, BD Biosciences). Briefly, intact smooth muscle strips were dissected from mouse corpus cavernosa and suspended in matrigel in a 48-well plate. Matrigel was allowed to polymerize at 37°C for 20–30 minutes. Strips were treated with either basal medium or nondiabetic-CM or diabetic-CM, or VEGF. Media were replaced with fresh media daily. Endothelial cells from corporeal sinusoids migrated into the three-dimensional matrix and formed endothelial sprouts followed by capillary-like tubes. Bright field images were acquired on day 5 at 4× or 10× magnification using an Olympus microscope. Angiogenic response was quantified by determining the length of the capillary tubes at four different locations around the central mass of tissue on the acquired image.

### Data Analysis and Statistics

Results are expressed as mean ± standard error of the mean (SEM) with “n” representing the number of donors. Either two or three replicates per donor were carried out in individual assays. Results were analyzed for statistical significance by GraphPad Prism software (GraphPad Software, CA, USA). Student's *t*-test was used for comparing levels of different cytokines and growth factors in CM and for comparing the effects of CM on proliferation and migration. BrdU incorporation was compared using the Kruskal-Wallis test. Student's *t*-test or two-way ANOVA with Neuman-Keuls post-test, as indicated, was used to analyse data from experiments in isolated arteries. Data from western blotting experiments were analyzed by the Mann-Whitney test. A *P* value of <0.05 was considered statistically significant.

## Results

### ECFCs and eEPCs were Infrequently Generated in Diabetic Individuals with Microvascular Complications

**Figure 1:**
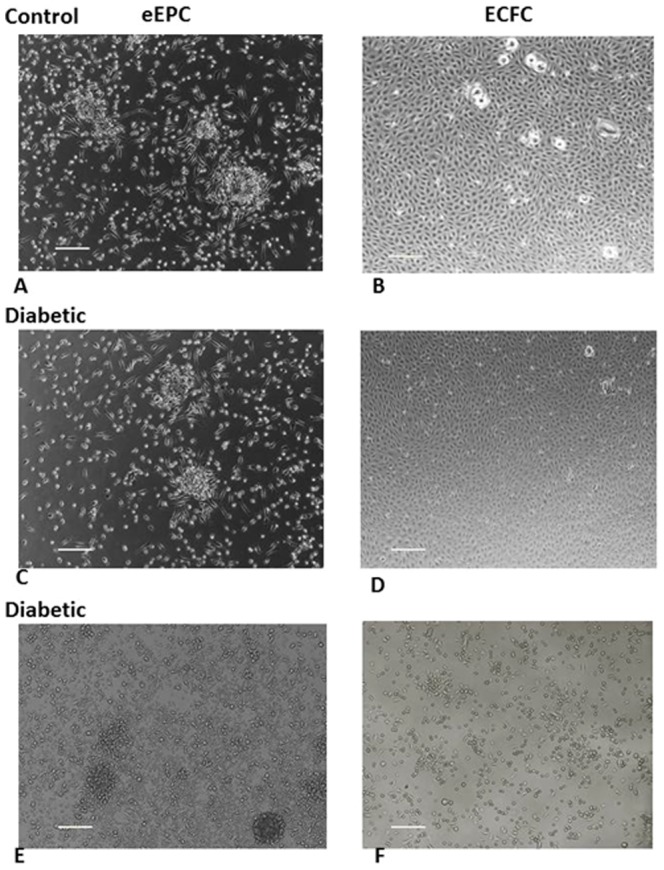
The representative images of eEPCs (A–C) and ECFCs (D–F) derived from peripheral blood MNCs obtained from healthy and diabetic individuals. Images **C** and **D**, and **E** and **F**, were derived from two different diabetic individuals, respectively, and showed inconsistency in the generation of eEPCs and ECFCs. Scale bar measures 200 microns.

Cohort 1 subjects (Table 1) were examined for their ability to grow eEPCs and ECFCs from peripheral blood. ECFCs and eEPCs were only obtained from 15% (three out of ten) of the diabetic individuals (Figure 1) while both types of cells could be grown from nearly 90% of the nondiabetic individuals (eight out of nine). Thus, all subsequent studies (Cohort 2) were pursued using CD34^+^ cells.

### Diabetic CD34^+^ Progenitors Demonstrate a Distinct Paracrine Secretory Profile

Due to the limited ability to expand ECFCs from diabetic subjects, we directed our efforts to the examination of CD34^+^ cell function, specifically their paracrine function which is critical for their reparative potential. Well-characterized DM individuals (Table 2) were compared to age and sex-matched nondiabetic subjects. Changes in the levels of factors in the CM of nondiabetic and diabetic cells are shown in Table 3. Levels of monocyte chemoattractant protein 1 (MCP1), granulocyte colony-stimulating factor (G-CSF), angiopoietin-1, FMS-like tyrosine kinase-3 ligand (Flt-3L), IL-3, IL-8, and IL-10 are unchanged (Table 3), while erythropoietin, basic nerve growth factor, transforming growth factor (TGFβ3), and tumor necrosis factor (TNFβ) are undetectable. Notably, the potent stem cell growth factors, stem cell factor (SCF), hepatocyte growth factor (HGF), and thrombopoietin (TPO) levels were significantly lower in diabetic-CM by 5- (*P*<0.0001), 9- (*P*<0.006), and 17-fold (*P*<0.0002), respectively, compared to nondiabetic-CM (Table 3). Secreted levels of GM-CSF were higher in diabetic compared to nondiabetic-CM (*P*<0.03).

In healthy tissue, exogenous progenitors may act by regulating acute inflammatory responses [39]. However, diabetic progenitors may aggravate the inflammatory response. To test this possibility we examined levels of IL-1β, IL-6, and TNF-α, all well-known pro-inflammatory factors. All three factors increased in diabetic-CM (*P*<0.05, *P*<0.01, and *P*<0.01, respectively) compared to nondiabetic-CM (Table 3). Previously, we demonstrated that TGF-β1 mRNA expression increased in CD34^+^ cells isolated from T2D, which implicated TGF-β1 in the reparative dysfunction of CD34^+^ cells [40]. Thus, we examined the levels of TGF-β1 and TGF-β2 in diabetic-CM and found that the levels were similar in diabetic-CM and nondiabetic-CM (Table 3). Interestingly, secretion of decorin, a natural antagonist of TGFβ1, was 19-fold lower in diabetic-CM (*P*<0.02, Table 3). Levels of IGF-1 and IGFBP-3 were higher in diabetic-CM (*P*<0.03 and *P*<0.008, respectively) compared to nondiabetic-CM (Table 3).

### Diabetic Progenitors Demonstrate Reduced Responsiveness to Hypoxia

**Figure 2:**
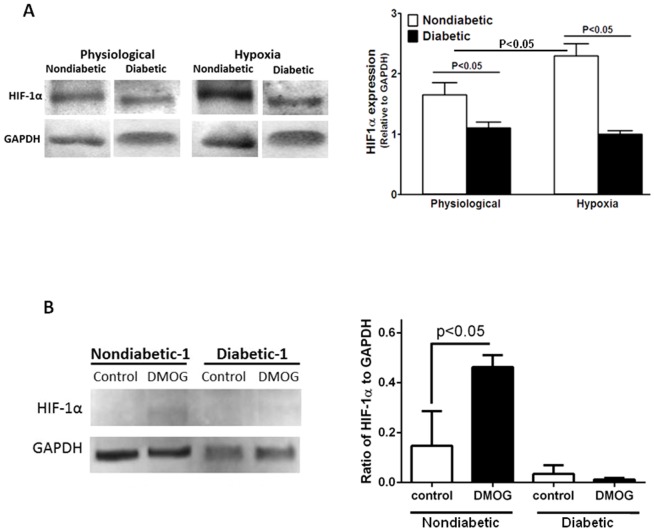
A, CD34^+^ cells of diabetic origin show hypoxia unresponsiveness. In hypoxic conditions, HIF1α expression, normalized to GAPDH, was enhanced in nondiabetic cells (*P*<0.02 vs. physiologic conditions). Diabetic cells did not show an increase in HIF1α expression in hypoxia and remained lower than nondiabetic cells (*P*<0.05, n = 5). **B**, Nondiabetic CD34^+^ cells showed increased expression of HIF1α (P<0.05, n = 5) in response to DMOG treatment while cells of diabetic origin did not respond.

Diabetic CD34^+^ cells do not respond to hypoxia-regulated factors, SDF-1 and VEGF[14]. To elucidate the mechanism involved in this dysfunction, we examined the expression of receptors for SDF-1α and VEGF, and activation of HIF1α, which regulates the expression of SDF-1α and VEGF when cells are exposed to hypoxia. Activation of HIF1α and expression of CXCR4, VEGFR1, and VEGFR2 under hypoxic conditions (pO_2_ = 5 mm Hg) were quantified. As expected in nondiabetic CD34^+^cells, exposure to hypoxia for 4 hours increased HIF1α expression (*P*<0.05, n = 5); however, diabetic cells did not show any change in expression and remained lower compared to nondiabetic cells (*P*<0.05, n = 5, Figure 2A). To further confirm the reduced response to hypoxia in diabetic CD34^+^ cells, cells were exposed to DMOG (Figure 2B) and HIF1α levels were determined. Similar to 4-hour hypoxia, DMOG treatment resulted in an increase in HIF1α in controls but not in diabetic cells.

**Figure 3:**
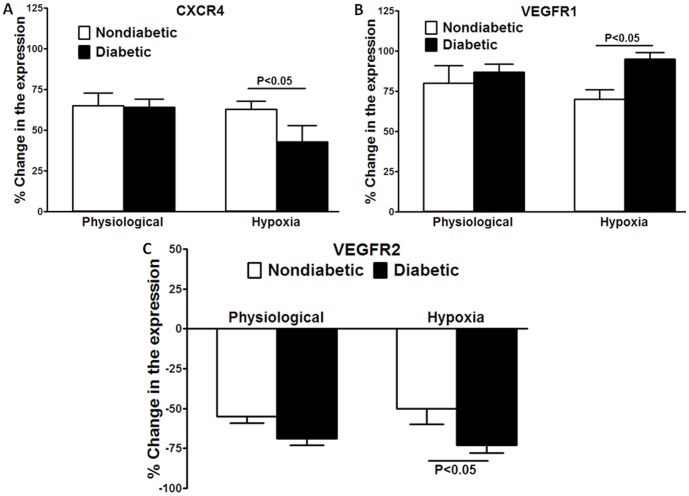
Surface expression of CXCR4, VEGFR1, and VEGFR2 in nondiabetic and diabetic CD34^+^ cells in hypoxic conditions (pO_2_ = 5 mm Hg) for 4 hours, expressed as percent change compared to the expression at zero time under standard incubator conditions (immediately prior to placing cells into a regulated oxygen environment). Physiologic concentrations of oxygen for the inner retina, pO2 = 40 mmHg and hypoxia, pO2 = 5 mmHg were achieved using oxygen controlled glove boxes attached via a central airlock with O2, CO2, N2, temperature and humidity control. A, Expression of CXCR4 was lower in diabetic cells following hypoxia compared to nondiabetic cells (*P*<0.05, n = 10). B, Expression of VEGFR1 was increased in diabetic cells following hypoxia compared to nondiabetic cells (*P*<0.05, n = 10). C, Expression of VEGFR2 was decreased in diabetic cells under hypoxic conditions compared to nondiabetic cells (*P*<0.05, n = 10).

Representative flow cytometry data of the expression of CXCR4, VEGFR1, and VEGFR2 are shown in Figure S1. Observed changes in the expression following 4-hour exposure to hypoxic conditions were expressed as percent change relative pre-exposure level. The expression of CXCR4 was decreased in diabetic CD34^+^cells while VEGFR1 expression was increased following exposure to hypoxia (*P*<0.05, n = 5, Figure 3). The expression of VEGFR2 decreased in hypoxic conditions compared to control levels. This decrease was similar in both diabetic and nondiabetic cells in physiological conditions, but the decrease was significantly greater in diabetic cells in hypoxic conditions (*P*<0.05, n = 5, Figure 3C). No changes were observed in the percent of receptor-expressing cells following exposure to hypoxia either in nondiabetic or diabetic cells (Table S1).

### Autocrine/Paracrine Proliferative and Migratory Functions are Impaired in Diabetic CD34^+^ Cells

**Figure 4:**
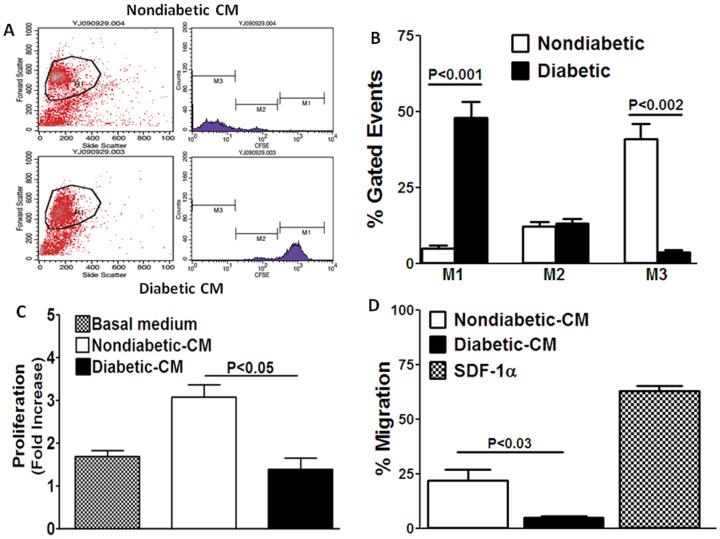
Paracrine proliferative and migratory functions of CM obtained from nondiabetic and diabetic CD34^+^ cells. **A**, Representative dot plots of CFSE-loaded CD34^+^ cells following 72 hour treatment with nondiabetic- or diabetic-CM. Number of events in the M1 region (high intensity region) represent the number of nonproliferating cells while those in the M3 region (low intensity region) represent proliferating cells. Events in the M2 region represent cells that are proliferating at lower rate. **B**, Proliferation was greater in cells treated with nondiabetic-CM compared to diabetic-CM (M3 region, **P<0.002), but was significantly lower in cells treated with diabetic-CM (M1 region, **P<0.001). **C**, BrdU incorporation in CD34^+^cells was enhanced by nondiabetic-CM by 3.2-fold compared to the basal medium. Diabetic-CM failed to induce BrdU incorporation (*P*<0.05). **D**, Nondiabetic-CM enhanced migration of CD34^+^ cells expressed as percent of untreated cells, which was approximately one third of the response produced by SDF-1α. Migratory response induced by diabetic-CM was significantly lower compared to the response produced by nondiabetic-CM (*P*<0.03).

CD34^+^ cells influence the function of neighboring CD34^+^cells and cells of the resident vasculature by stimulating proliferation and migration of these cells via autocrine/paracrine mechanisms [27]. The effects of CM on proliferation and migration of CD34^+^ cells obtained from nondiabetic individuals was evaluated next. After 72 hours of exposure to nondiabetic-CM, nondiabetic CD34^+^ increased their proliferation as determined by division tracking cytometry using carboxyfluorescein diacetate succinimidyl ester (CFSE). Increased proliferation was shown by the higher number of events in the low intensity region M3 and lesser number of events in the high intensity region M1 (Figure 4A and 4B). In contrast, treatment with diabetic-CM did not support proliferation of these cells, as indicated by lower number of events in the M3 region (*P*<0.001, n = 4) and higher number of events in the M1 region (*P*<0.001, n = 4) (Figure 4B). These results were further confirmed by evaluating BrdU incorporation. This was quantitatively expressed as fold-increase compared to that observed in the presence of 10 μM mitomycin C, which is known to inhibit cell proliferation. In the basal medium, CD34^+^ cells proliferated 1.6-fold, which was significantly enhanced to 3.2-fold by nondiabetic-CM (*P*<0.05, n = 4) (Figure 4C). Diabetic-CM enhanced BrdU incorporation by only 1.3-fold, which was significantly lower than the effect of nondiabetic-CM (*P*<0.05, n = 6) (Figure 4C).

We next tested whether the diabetic-CM could induce a migratory response in nondiabetic CD34^+^ cells as suggested by Krenning et al [27]. SDF-1α induced 63±3% (n = 6) (Figure 4D) increase in migratory response in nondiabetic-CD34^+^cells. Nondiabetic-CM stimulated migration of nondiabetic CD34^+^cells (22±5%, n = 4) (Figure 4D), which was about one third of the response produced by SDF-1α. The response to diabetic-CM was 5-fold lower than nondiabetic-CM (5±0.7%, P<0.03, n = 4) (Figure 4D). These observations strongly suggest that autocrine/paracrine modulation of CD34^+^cell function is impaired in diabetes.

### Paracrine Vasodilatory Function is Impaired in Diabetic CD34^+^ Cells

**Figure 5:**
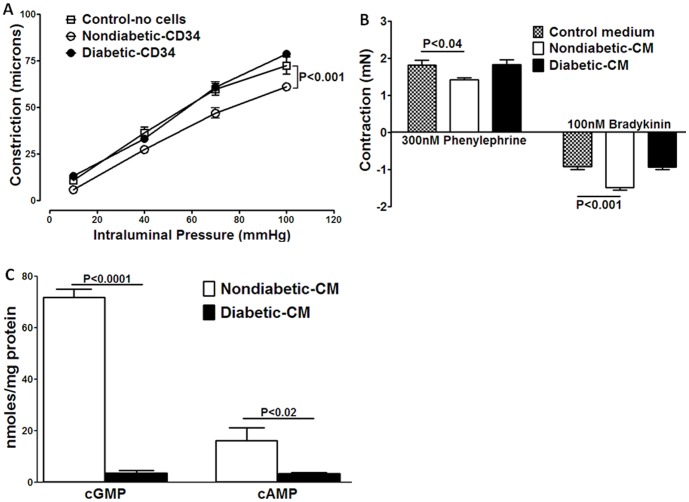
Paracrine functions of nondiabetic and diabetic CD34^+^ cells and CM derived from these cells in rat arterioles. **A**, Constriction of rat posterior cerebral arterioles with increased intraluminal pressure was lower when nondiabetic cells were present intraluminally compared to constriction without cells (*P*<0.001, two-way ANOVA). Constriction was unaltered by diabetic cells. **B**, Contraction to 300 nM phenylephrine was decreased by nondiabetic-CM compared to the control medium (*P<0.04), but diabetic-CM had no effect. Relaxation to 100 nM bradykinin was potentiated by nondiabetic-CM compared to the control medium (**P<0.001) and was not altered in the presence of diabetic-CM. **C**, Nondiabetic-CM stimulated formation of cGMP and cAMP in the intact arterial segments, and the response produced by diabetic-CM was significantly lower [*P*<0.0001 (cGMP) and *P*<0.02 (cAMP), n = 5].

Vasoreparative function of cells involves the ability of circulating angiogenic cells to dilate vasculature in areas of ischemic injury to enhance blood flow and increase delivery of reparative cells. Our hypothesis predicts that diabetic cells lack this paracrine vasodilatory effect. To test this possibility, we evaluated the effect of CD34^+^ cells on healthy rat posterior cerebral arteries that are highly constrictive to intraluminal pressure and are known to mediate autoregulation of blood flow [41]. The use of diabetic arteries was not considered feasible as the vessels are dysfunctional, characterized by impaired endothelium-dependent dilatation and increased constriction [42]–[44]. The intraluminal presence of nondiabetic CD34^+^ cells (1×10^5^) attenuated the pressure-induced constriction compared to that in the absence of cells (Figure 5A) (*P*<0.001, n = 5, two-way ANOVA). Post-test analysis determined that the significant differences were at pressures of 70 and 100 mm Hg (*P*<0.001). In the presence of diabetic CD34^+^ cells (1×10^5^), constriction was not affected at any pressure (Figure 5A) compared to the control.

Next, the effect of paracrine factors in the diabetic-CM and nondiabetic-CM on the vascular reactivity was evaluated in wire-mounted mesenteric arteries. Contraction to phenylephrine and relaxation to bradykinin were evaluated in the presence of CM. In the presence of nondiabetic-CM, contraction to submaximal concentrations of phenylephrine (300 nM) was significantly attenuated (*P*<0.04, n = 4) compared to the control medium (Figure 5B). However, this attenuation in contraction was not observed in the presence of diabetic-CM. Relaxation to submaximal concentrations of bradykinin (100 nM) was enhanced in the presence of nondiabetic-CM compared to the control medium (*P*<0.001), and this potentiation was not observed in the presence of diabetic-CM (Figure 5B). In agreement with this observation, nondiabetic-CM stimulated formation of cGMP and cAMP in the intact arterial segments while the levels generated by diabetic-CM were significantly lower [*P*<0.0001 (cGMP) and *P*<0.02 (cAMP), n = 5, Figure 5C)]. These results suggest that the paracrine factors produced by nondiabetic cells are capable of attenuating agonist-mediated vasoconstriction and potentiating agonist-induced vasodilation by enhancing cGMP and cAMP levels in the vasculature and that this paracrine function was dramatically reduced in diabetic CD34^+^ cells.

### Paracrine Angiogenic Function is Impaired in Diabetic CD34^+^ Cells

**Figure 6:**
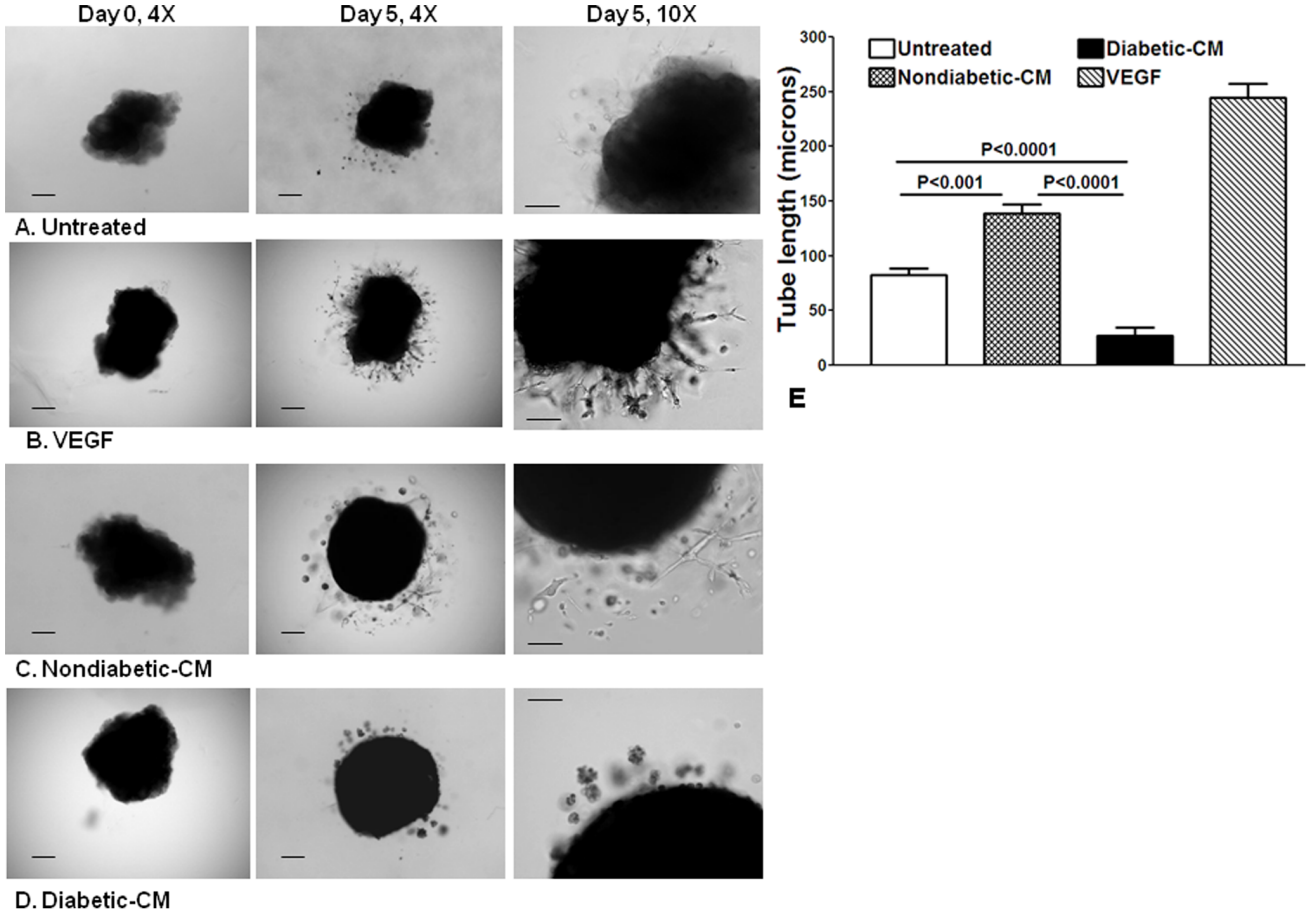
Paracrine angiogenic functions of CM obtained from nondiabetic and diabetic CD34^+^ cells. Mouse corporeal strips were suspended in matrigel and treated with different media for 5 days. Bright-field images were obtained at 4× or 10× magnification. Day 0 shows strips in liquid matrigel before polymerization at 37°C. **A**, Representative images of corporeal strips treated with basal medium show migration of endothelial cells into matrigel that tend to form poorly defined tubular structures at different time intervals. **B**, Treatment of corporeal strips with 100 nM VEGF stimulated migration of endothelial cells into the matrigel, with endothelial sprouting, and formation of tubular structures. **C**, Treatment of corporeal strips with nondiabetic-CM stimulated migration of endothelial cells into the matrigel that tended to form sprouts and tubular structures. **D**, Treatment of corporeal strips with diabetic-CM stimulated minimal migration of endothelial cells and cells tended to form clumps, which failed to form defined tubular structures. Scale bar measures 100 microns. **E**, Quantification of angiogenic response determined by tube length (microns): Response to the non-diabetic CM is higher than the response observed in the untreated strips (*P<0.001*, n = 5). Response to diabetic-CM was lower compared to the untreated (*P<0.0001*, n = 5) or nondiabetic-CM-treated strips (*P<0.0001*, n = 5).

The pro-angiogenic function of paracrine factors was evaluated in an *in vitro* angiogenesis assay using corporeal vascular tissue explants in matrigel culture. Formation of capillary-like tubular structures with sprouting endothelial cells is an indicator of angiogenic function. In the presence of basal medium, few endothelial cells migrated from the tissue sinusoids and formation of clear capillary tubes was minimal after five days of matrigel culture (Figure 6A). In the presence of VEGF (100 nM), several sprouting endothelial cells were observed, which formed an extensive formation of capillary tubes (Figure 6B). Nondiabetic-CM stimulated migration of endothelial cells into the matrix, that tended to form clear capillary tubes, suggesting a pro-angiogenic potential (Figure 6C). However, in the presence of diabetic-CM, very few cells migrated into the matrix, which tended to form clumps and did not form capillary structures (Figure 6D). Quantification of angiogenic response as measured by capillary tube length was lower in the diabetic-CM treated compared to nondiabetic-CM treated tissues (*P<0.0001*) or compared to the untreated (*P<0.0001*) (Figure 6E).

## Discussion

In this study, we report for the first time several highly novel findings. In diabetic individuals with microvascular complications, the ability to establish ECFCs in culture is markedly reduced. Martin-Ramirez et al has shown that success rate of generation of ECFCs are ∼80% from normal donors [45], whereas we found a failure rate of ∼85% in diabetic donors. Our results would thus suggest that it would be critical to establish ECFCs from a diabetic individual early in their disease when their peripheral blood retained sufficient numbers of these progenitor cells. The ECFC populations would require cell banking for later use by the individual when an autologous cell therapy would be considered for treatment of vascular complications.

While we have established that CD34^+^ cells may be a more practical source of cells as no ex vivo expansion is required, these cells are largely dysfunctional and thus their utility as a cell therapy is also limited. Ex vivo manipulation to restore CD34^+^ cell function would be required prior to reinfusion.

Our study also supports that human CD34^+^ cells from healthy individuals release a unique repertoire of growth factors and cytokines that demonstrate pro-proliferative, pro-migratory, pro- or anti-inflammatory and vasodilatory properties. Paracrine vasodilatory effects are mediated by directly enhancing generation of cGMP and cAMP in the vasculature. In contrast, diabetic-CD34^+^ cells showed a marked decrease in the release of the proangiogenic factors SCF, HGF, and TPO but an increase of the pro-inflammatory factors GM-CSF, IL1β, IL6, and TNFα. Diabetic-CD34^+^ cells exhibited impaired paracrine effects on vascular reactivity, tissue angiogenesis, proliferation, and migration. Furthermore, sensitivity to hypoxia was impaired in diabetic CD34^+^cells.

SCF, HGF, and TPO are pro-angiogenic, regulate proliferation, differentiation and migration of CD34^+^ cells, and stimulate vascular endothelial function.[46]–[50] Decreased release of these factors by diabetic CD34^+^cells was reflected in impaired proliferative and angiogenic responses of the diabetic-CM. Furthermore, this functional impairment was not compensated for by other pro-proliferative factors such as IL-3, Flt3L, or GM-CSF, whose levels were either unchanged or enhanced.

Higher levels of pro-inflammatory factors, such as IL1β, IL6, and TNFα, likely contributed to the impaired angiogenic function observed in diabetic cells. IL6 may promote an anti-angiogenic effect via inducing pathological vascular remodeling by activating bone morphogenetic protein receptor type II (BMPR-II) signaling.[51], [52] GM-CSF, a hematopoietic cytokine, triggers the inflammatory cascade and, at relatively higher concentrations, stimulates mobilization of bone marrow-derived cells into the circulation [53]. While an inflammatory response is required to initiate an angiogenic reaction,[39] a prolonged inflammatory stimulus typically impedes the angiogenic process.

The vascular repair process that originates from the bone marrow is triggered by cytokines, growth factors, and other inflammatory factors. In diabetes, an inadequate repair process can lead to a progressive amplification of the inflammatory signals due to the lack of a negative feedback inhibition. These signals in turn contribute to the exaggerated inflammatory reaction, further damaging the arterial wall and impairing the process of vascular repair.[54], [55]

The present study suggests that IL-1β and TNF-α may play a major role in this amplification of inflammation, and that high levels of the anti-inflammatory factor, TGF-β, serve to counterbalance this inflammatory response at the expense of inducing growth inhibition in CD34^+^ cells.[40] Moreover, even the higher expression of VEGF by diabetic-CD34^+^cells can represent an indicator of exaggerated inflammation rather than an indicator of hypoxia in the absence of nitric oxide (NO). VEGF promotes a pro-inflammatory cascade when intracellular NO levels are low and leads to enhanced proliferation, plus migration of endothelial cells, smooth muscle cells, and macrophages[56].

Recently, an elegant study by Schatteman et al [39] showed decreased secretion of TNFα and MCP1 in murine BM-Lin^-^ cells of type-2 diabetic (*Lepr^db^*) origin compared to wild-type mice, with no change in the secretion of IL1β, IL4, and IL6. Furthermore, secretion of IL2, IL5, IL10, IL12, and GM-CSF was not observed in this study. IL6 expression was not significantly changed in STZ-diabetic Lin^-^Sca1^+^cKit^+^ cells compared to wild type mice.[57] These studies, when compared to the current study using human CD34^+^cells, suggest differences in the secretion profile of murine bone marrow-derived cells vs. human peripheral blood CD34^+^ cells.

Recently, we have shown that TGFβ1 mediates vasoreparative dysfunction in CD34^+^cells from diabetics and that the transient blockade of the expression of TGFβ1 or PAI-1, the main gene target of TGFβ, restores vasoreparative function. In our previous study, we only examined changes in TGFβ mRNA levels of CD34^+^ cells and did not examine the secretion pattern of TGFβ1.[40] In the present study, we show that the secreted levels of TGFβ1 were similar in nondiabetic and diabetic cells. However, levels of decorin were significantly reduced in diabetic-CM. Decorin modulates TGFβ1 function by regulating its bioavailability and intracellular signaling, and it is considered a natural antagonist of TGFβ1.[58], [59] Reduced decorin levels would therefore result in prolonged TGFβ1-mediated effects of diabetic CD34^+^ cells.

IGF1 protects against endothelial dysfunction, atherosclerosis, and cardiac ischemic damage,[60] and was shown to enhance proliferation of CD34^+^ and mesenchymal progenitor cells.[61] In a similar manner, we and others have shown that IGFBP-3 has IGF1-independent vascular protective effects by enhancing vasoreparative functions of CD34^+^ cells and their mobilization from bone marrow into the circulation.[62] Increased secretion of these two factors by diabetic CD34^+^cells may represent a compensatory effect but clearly is not sufficient to rescue their reparative function.

Previously, we have shown that the diabetic cells do not respond to hypoxia in in vivo models of retinal ischemia [15] and we have also demonstrated that the diabetic CD34^+^cells do not migrate towards hypoxia regulated factors, SDF-1 and VEGF [63]. In the current study, we have observed that the diabetic cells did not respond to hypoxia by up-regulating HIF1α that was accompanied by significant changes in the receptors' expression in response to hypoxia. CXCR4 expression was decreased in diabetic cells following exposure to hypoxia; whereas, VEGFR1 expression was increased. The observation of a decrease in VEGFR2 surface expression was unexpected but highly reproducible, while it is difficult to explain. We postulate that there is internalization of VEGFR2 when cells are transferred from standard incubator conditions to either hypoxia or physiologic conditions as occurred in this study. We and others have shown that VEGFRs are internalized either for degradation or translocated to other parts of the cell [64]–[66].

We further demonstrate autocrine/paracrine functions of CD34^+^ cells in inducing migration and endothelial sprouting and capillary tube formation from tissue-resident endothelial cells. It would be difficult to attribute these beneficial functions to one or another paracrine factor produced by these cells. Rather, it could be a net effect of the combination of these factors. The migration studies were designed to examine the cumulative effects of the molecules in the CM, rather than specific factors. The conclusions from migration studies, however, must be considered within the context of the technical limitations of the study design. The isolated CD34^+^ cells were maintained in a serum-free environment in order to exclude the contribution of factors in the serum, and the cells were kept in static (no flow) conditions. Nonetheless, the “net” effect of the CM is greatly impaired in diabetes. Awad et al[24] reported that obesity and diabetes, together, convert the pro-angiogenic phenotype to an anti-angiogenic phenotype in CD34^+^ cells. These findings can largely be explained by paracrine release of anti-angiogenic factors by CD34^+^ cells of obese/diabetic origin.

Our study provides direct evidence for the paracrine vascular effects of CD34^+^ cells and provides compelling evidence for paracrine dysfunction of diabetic CD34^+^ cells on the vasculature. Significant dilatory effects were observed by the intraluminal application of CD34^+^ cells and by their CM in arteries. Signaling mechanisms involving cGMP and cAMP are major pathways that mediate vasodilation in response to the majority of endogenous vasodilators. According to our study, CD34^+^ cells promote vasodilation by attenuating vascular tone in response to intraluminal pressure. In agreement, nondiabetic-CM attenuated constriction to phenylephrine, potentiated dilation to bradykinin, and increased generation of cGMP and cAMP levels in the vascular wall. Dilation of arterioles in the peri-ischemic areas accelerates the process of vascular repair by enhancing blood flow, thereby increasing the delivery of reparative cells and transport of circulating vasoactive substances. These beneficial vascular effects were impaired in diabetic CD34^+^ cells.

It is presently thought that diabetic CD34^+^ cells will likely need *ex vivo* modification prior to their utilization in any treatment strategy. Assessment of their secretory profiles may represent a critical way to evaluate their therapeutic utility prior to use as a cell therapy. Thus, evaluation of their paracrine secretory profile may be the key component for the optimization of these cells as a vasoreparative population. Previously, overexpression of Akt/PKB in mesenchymal stem cells was shown to enhance their cardiac reparative function by paracrine mechanisms and was used to assess therapeutic utility; although, the cells studied were derived from normal mice.[67], [68]

In conclusion, this study suggests that the ex vivo expanded population, ECFCs may be limited as a viable therapeutic option, unless these cells are obtained early in the disease prior to the development of microvascular complications. While CD34^+^ cells can be easily obtained from diabetics with complications, these cells have marked paracrine dysfunction and an altered response to hypoxia that likely mediates much of this dysfunction and limits there therapeutic utility. *Ex vivo* strategies are now being developed to correct autologous CD34^+^ cell dysfunction prior to reintroducing these cells to the patient for treatment of vascular complications in diabetic individuals. Furthermore, our results suggest that hypoxia responsiveness and measurement of key secreted factors of CD34^+^ cells may provide the critical information to valid the subsequent in vivo function of these cells. Use of these additional parameters may provide a more rational basis for determining the therapeutic utility of CD34^+^ cells and would likely improve outcomes of clinical trials using autologous cells.

## Supporting Information

Figure S1 **Representative flow cytometry data of the expression of CXCR4, VEGFR1, and VEGFR2 in A, nondiabetic CD34^+^ cells and B, diabetic cells at zero hour following 4 hour exposure to physiological (pO_2_ = 40 mm Hg) or hypoxic (pO_2_ = 5 mm Hg) environments.** Red and black tracings in each plot represent cell populations labeled with the Alexa Fluor 488-conjugated antibody of the receptor as indicated or its isotype control, respectively.
(TIF)

Table S1 **Hypoxia-induced changes in the number of receptor-expressing CD34^+^ cells, expressed as % of CD34^+^ cells, from nondiabetic and diabetic individuals.**
(DOCX)
