# Correction: Vasoreparative Dysfunction of CD34^+^ Cells in Diabetic Individuals Involves Hypoxic Desensitization and Impaired Autocrine/Paracrine Mechanisms

**DOI:** 10.1371/journal.pone.0103913

**Published:** 2014-07-23

**Authors:** The PLOS ONE Staff

Three of the author names appear incorrectly in this published article.

The fourth author’s name is missing a space. The correct name is Sergio Li Calzi.

The fifth author’s name is spelled incorrectly. The correct name is Chandra Jadhao.

The sixth author’s initials are incorrect in the author contributions. The correct author contributions for "Performed the experiments" are: YPRJ SH QQ.

The correct citation is:

Jarajapu YPR, Hazra S, Segal M, Li Calzi S, Jadhao C, et al. (2014) Vasoreparative Dysfunction of CD34^+^ Cells in Diabetic Individuals Involves Hypoxic Desensitization and Impaired Autocrine/Paracrine Mechanisms. PLoS ONE 9(4): e93965. doi:10.1371/journal.pone.0093965
