# Supplementary figures and images for: Vasoreparative Dysfunction of CD34+ Cells in Diabetic Individuals Involves Hypoxic Desensitization and Impaired Autocrine/Paracrine Mechanisms
Source: PLoS One. 2014 Apr 8;9(4):e93965. doi: 10.1371/journal.pone.0093965 (PMC3979711; doi:10.1371/journal.pone.0093965)

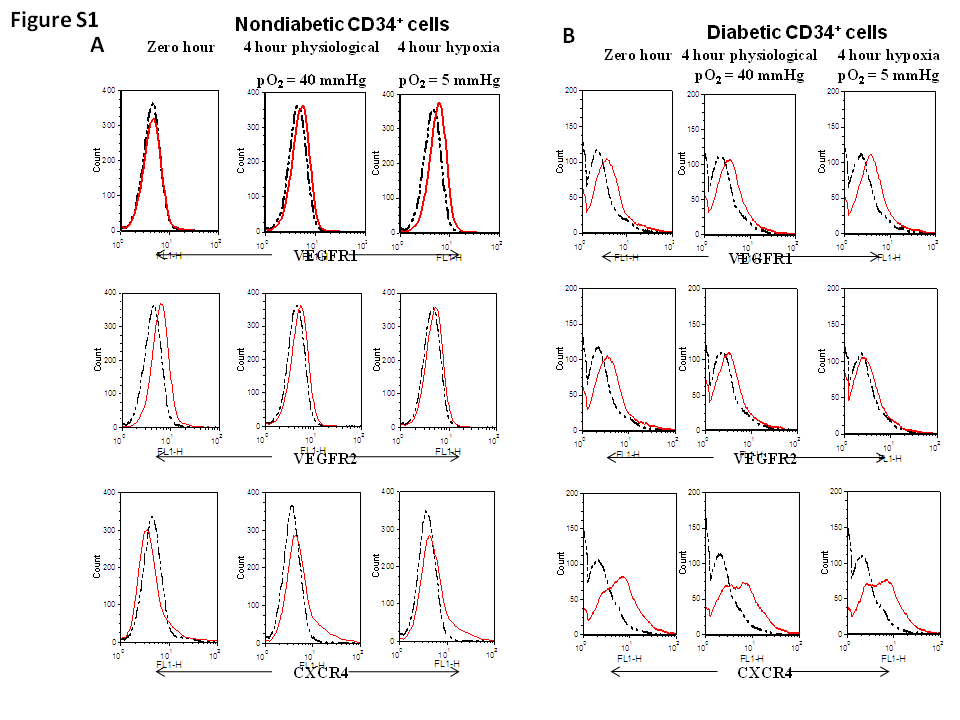

Supplement: Figure S1 — Representative flow cytometry data of the expression of CXCR4, VEGFR1, and VEGFR2 in A, nondiabetic CD34+ cells and B, diabetic cells at zero hour following 4 hour exposure to physiological (pO2 = 40 mm Hg) or hypoxic (pO2 = 5 mm Hg) environments. Red and black tracings in each plot represent cell populations labeled with the Alexa Fluor 488-conjugated antibody of the receptor as indicated or its isotype control, respectively. (TIF) [file pone.0093965.s001.tif]
