# Supplementary material for: Vasoreparative Dysfunction of CD34+ Cells in Diabetic Individuals Involves Hypoxic Desensitization and Impaired Autocrine/Paracrine Mechanisms
Source: PLoS One. 2014 Apr 8;9(4):e93965. doi: 10.1371/journal.pone.0093965 (PMC3979711; doi:10.1371/journal.pone.0093965)
Supplement: Table S1 — Hypoxia-induced changes in the number of receptor-expressing CD34+ cells, expressed as % of CD34+ cells, from nondiabetic and diabetic individuals. (DOCX) [file pone.0093965.s002.docx]

**Table-S1. Hypoxia-induced changes in the number of receptor-expressing CD34^+^ cells, expressed as % of CD34^+^ cells, from nondiabetic and diabetic individuals.**

**Receptor Nondiabetic Diabetic**

**Physiological Hypoxia Physiological Hypoxia**

**CXCR4 59±6 62±7 55±4 58±5**

**VEGFR1 24±3 26±4 27±5 30±4**

**VEGFR2 33±5 37±4 31±4 35±6**
